# Light Scattering Properties Vary across Different Regions of the Adult Mouse Brain

**DOI:** 10.1371/journal.pone.0067626

**Published:** 2013-07-09

**Authors:** Saif I. Al-Juboori, Anna Dondzillo, Elizabeth A. Stubblefield, Gidon Felsen, Tim C. Lei, Achim Klug

**Affiliations:** 1 Department of Electrical Engineering, University of Colorado Denver, Colorado, United States of America; 2 Department of Physiology and Biophysics, University of Colorado School of Medicine, Aurora, Colorado, United States of America; University of Zurich, Switzerland

## Abstract

Recently developed optogenetic tools provide powerful approaches to optically excite or inhibit neural activity. In a typical *in-vivo* experiment, light is delivered to deep nuclei via an implanted optical fiber. Light intensity attenuates with increasing distance from the fiber tip, determining the volume of tissue in which optogenetic proteins can successfully be activated. However, whether and how this volume of effective light intensity varies as a function of brain region or wavelength has not been systematically studied. The goal of this study was to measure and compare how light scatters in different areas of the mouse brain. We delivered different wavelengths of light via optical fibers to acute slices of mouse brainstem, midbrain and forebrain tissue. We measured light intensity as a function of distance from the fiber tip, and used the data to model the spread of light in specific regions of the mouse brain. We found substantial differences in effective attenuation coefficients among different brain areas, which lead to substantial differences in light intensity demands for optogenetic experiments. The use of light of different wavelengths additionally changes how light illuminates a given brain area. We created a brain atlas of effective attenuation coefficients of the adult mouse brain, and integrated our data into an application that can be used to estimate light scattering as well as required light intensity for optogenetic manipulation within a given volume of tissue.

## Introduction

Manipulating neural function with light is becoming an increasingly important technique. This is particularly important for the recently emerging field of optogenetics, which provides powerful tools to either activate or suppress neural activity with light at a relatively fast time scale (e.g. [1]–[3]). Controlling neuronal firing with light has opened up not only a number of exciting new avenues to study neural circuits, but also treatment options for a number of medical conditions such as Parkinson’s disease and certain forms of blindness [4]–[6].

For experiments using cell cultures or brain slices, the precise and reliable delivery of light to the neurons to be manipulated is relatively simple and is typically achieved by attaching a suitable light source to a microscope, and subsequently delivering light stimuli with the desired parameters directly to the neural tissue. For *in-vivo* experiments, however, light delivery to deep brain areas is much more challenging. Typically, investigators use stereotaxic methods to place an optical fiber just above the brain area to be illuminated, such that light exiting the fiber effectively illuminates the tissue below the fiber tip [7].

Depending on the optical properties of the specific tissue, light emitted from the fiber tip propagates deeper or less deep through the tissue, with neurons more distant from the fiber tip receiving higher or lower light intensities. All light sensitive molecules (such as the various opsins typically used in optogenetic experiments, but also caged compounds and fluorescent dyes) have a threshold of activation, (for the purpose of this publication defined in practical terms as the minimum light intensity required to effectively trigger the desired photochemical reaction). Therefore, light sensitive molecules can only be activated within a certain maximum distance from the light source, and this distance depends on both the optical properties of the tissue and the activation threshold of the molecule used in the experiment. Most studies involving delivery of light to deep brain areas assume, for simplicity, that all brain tissue scatters light in the same way, i.e. different brain areas behave similarly if not identically as far as light propagation in the tissue is concerned [8]–[10]. However, some brain areas consist primarily of cell bodies while others consist primarily of fibers, and some brain areas appear darker while others appear lighter when observed under a microscope with transmitted light, suggesting differences in optical properties between different brain areas.

The main goal of this study was to measure light propagation and light scattering in different brain areas. Significant differences in these properties between different brain areas would indicate that specific knowledge about the brain area to be manipulated is required for the appropriate design of experimental manipulations. A secondary goal of the study was to establish a database of light scattering values for different areas of the mouse brain that could be used as a reference in future experiments.

Our experimental approach was to use sections of fresh brain tissue in combination with light emitting optical fibers that were advanced through the tissue to precisely measure light scattering properties. The results presented here are supplemented by an online light scattering mouse brain atlas and a computer program. These tools are intended to aid an investigator in determining the required light intensity to be delivered for successful optogenetic manipulation.

## Materials and Methods

### Optical Fiber Assembly

Three different optical fiber assemblies were used for the measurements. All three assemblies consisted of 100 µm core diameter optical fibers (UM22–100, Thorlabs, Newton, NJ) attached to 453 nm (blue), 528 nm (green), and 940 nm (near infra-red) LEDs, respectively. All LEDs were purchased from Digikey (Thief River Falls, MN). The optical fiber was lined up with its respective LED using two precision manipulators. The alignment was carefully done to obtain maximum optical throughput but avoiding crashing the fiber tip into the LED. UV optical epoxy was used to set the optical fiber in place and to secure the alignment between the LED and the optical fiber. In each case, the LED-optical fiber assemblies were powered by a Mightex LED power supply (SLB-1200-1), allowing the optical power output to be adjusted by changing the electrical current running through the LEDs.

### Ethics Statement

All animal procedures were approved by the Institutional Animal Care and Use Committee (IACUC) of the University of Colorado Medical Campus (Permit number B-88412(05)1D. Furthermore, all applicable laws and regulations, as well the PHS Policy were strictly followed.

### Animal Subjects

34 male and female C57BL/6J mice were used in these experiments. All animal procedures were approved by the University of Colorado Institutional Animal Care and Use Committee, and were conducted in accordance with National Institutes of Health standards on humane treatment of laboratory animals.

### Slice Preparation

Coronal and sagittal brain slices were prepared from six to eight weeks old mice. Animals were briefly anesthetized via isoflurane inhalation (IsoFlo, Abbott Laboratories, USA), and decapitated. The brain was dissected out under ice-cold dissection Ringer containing either (in mM): Ringer 1∶125 NaCl, 2.5 KCl, 1 MgCl_2_, 0.1 CaCl_2_, 25 glucose, 1.25 NaH_2_PO_4_, 25 NaHCO_3_, 0.4 ascorbic acid, 3 myo-inositol, and 2 pyruvic acid; or Ringer 2∶200 sucrose, 1.25 NaH2PO4, 26 NaHCO3, 10 glucose, 3.5 KCl, 7 MgCl, 1.5 ascorbic acid (all chemicals from Sigma). Sections of 600 µm were cut with a vibratome (VT1000S, Leica), transferred to an incubation chamber containing extracellular solution [ECS; containing (in mM) 125 NaCl, 2.5 KCl, 1 MgCl_2_, 2 CaCl_2_, 25 glucose, 1.25 NaH_2_PO_4_, 25 NaHCO_3_, 0.4 ascorbic acid, 3 myo-inositol, and 2 pyruvic acid, all chemicals from Sigma] and bubbled with 5% CO_2_-95% O_2_. Slices were incubated in ECS for 15–30 minutes at 37°C and then cooled down to room temperature. All measurements were obtained within 2–3 h of slicing.

### Imaging

**Figure 1:**
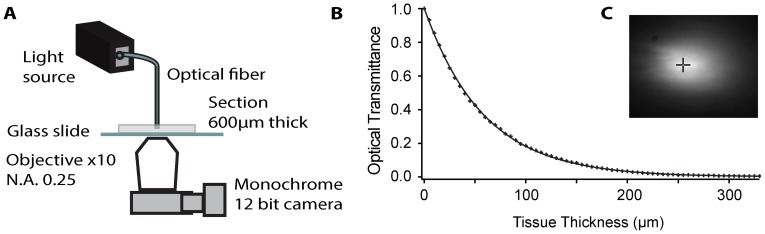
The experimental approach and sample data. A: Basic experimental setup with the punch-through method. On an inverted microscope, an optical fiber was placed on a section of brain tissue such that light from the fiber would pass through the tissue and subsequently be imaged by an objective attached to a CCD camera. B: Optical transmittance as a function of tissue thickness. As the optical fiber was advanced through the section of brain tissue and repeated images such as the one in 1C were taken, the decrease in optical transmittance as a function of tissue thickness could be evaluated. The single measurements (“+” symbols) represent transmittance of blue light (453 nm) through a section of PPT at various thicknesses, while the solid line represents an exponential fit. C: An example of an original image captured by the CCD camera, showing light emitted from an optical fiber after it passed though a section of brain tissue.

After the incubation period, a slice was placed into a measurement chamber and continuously superfused with bubbled extracellular solution for the duration of the experiment. The measurement chamber was then positioned on an inverted microscope (Nikon Diaphot 200, Nikon Corp., Japan) in which the standard transmitted light source was replaced by an assembly consisting of a three-axis manual micromanipulator (Narishige model MM-3), a calibrated piezo driven one axis micromanipulator (Model 8302 Picomotor Actuator, Newport, Irvine, CA), and a custom made optical fiber holder to hold one of the three fiber/LED assemblies in place. The output end of the optical fiber was placed directly onto the surface of the brain slice under the guidance of a CCD camera using macro optics, such that the emitted light was facing the brain section and the microscope’s objective (EF 10x, N.A. 0.25, Leitz Wetzlar, Germany). The light was then captured by a monochromatic 12 bit camera (Mightex CCE-B013-U) attached to the microscope via the camera port (see Fig. 1A for a sketch of the setup).

Exposure time and the irradiance of the optical fiber (I_0_) were adjusted to optimally utilize the Mightex camera dynamic range throughout the entire data set. Subsequently, the fiber was lowered into the slice in 5 µm steps using the precision piezo micromanipulator, starting from the surface of the section and ending at a depth of 500 µm. Control experiments measured the forces applied to the tissue section by the advancing glass fiber. The rationale of these experiments was that a glass fiber that cuts through the tissue easily and cleanly would apply little force onto the tissue section. By contrast, a glass fiber that does not cut through the section but rather push onto it and compress the tissue should apply significantly more force onto the tissue. We measured the forces that advancing glass fibers apply onto tissue sections with a standard micro balance (Mettler Toledo Series XP). These experiments determined that the maximum force applied to the tissue that was measured over repeated trials was less than 200 µN (n = 3 penetrations, maximum force measured over these three trials was 181 µN). This force is comparable to the forces created by an advancing sharp microelectrode (<200 µN). We thus concluded that lowering the fiber into the tissue caused the fiber tip to slice through, rather than squish the tissue together, such that measurements at many different tissue thicknesses could be taken reliably from the same tissue section at precisely controlled depths (referred to as “fiber punch-through method”).

A custom computer program running under Labview (National Instruments, Austin, TX) controlled the piezo micromanipulator and the Mightex camera. Images taken at different steps were stored for further data analysis. An example of such an image is shown in figure 1C. The data was extracted from images by locating the pixel representing the fiber center, and collecting that pixel 12 bit gray scale value for the digitized optical irradiance *I(z)*, this process was repeated for each image. *I(z)* was normalized to *I_0_* to obtain the optical transmittance *T(z),* which was then fitted by a single exponential function (Fig. 1B), according to the modified Beer-lambert law (see below) to extract the effective attenuation coefficient *µ_eff_* of the measured neural target.

### Data Collection and Analysis

Using this procedure, data were collected from several brain areas: Medial Nucleus of the Trapezoid Body (MNTB), Ventral Nucleus of the Trapezoid Body (VNTB), Lateral Superior Olive (LSO); pedunculopontine tegmental nucleus (PPT), superior colliculus (SC), Olfactory bulb (OB), and the cerebellar cortex molecular layer. The brain regions were chosen because previous knowledge suggested that they would represent a wide range of scattering coefficients, but also to perform control experiments for future optogenetics manipulations. The MNTB, VNTB and LSO were measured with three wavelengths of light, while all other nuclei were measured with one wavelength, (see table 1 for summary). One set of measurements was performed per brain nucleus per hemisphere.

**Table 1:** Brain areas that were measured with three different wavelengths, and sample size (the unit of the effective attenuation coefficient is 1/mm).

Brain Area	Effective Attenuation Coefficient µ_eff_ (1/mm)		
	λ_1_ = 453 (nm)	λ_2_ = 528 (nm)	λ_3_ = 940 (nm)
**MNTB**	18.16 (n = 11)	15.86 (n = 9)	13.86 (n = 8)
**VNTB**	19.96 (n = 6)	17.69 (n = 7)	14.39 (n = 7)
**LSO**	17.92 (n = 4)	15.91 (n = 7)	14.01 (n = 6)
**PPT**	15.26 (n = 10)		
**SC**	13.91 (n = 10)		
**CA3**	19.12 (n = 8)		
**Cerebellum**	9.76 (n = 8)		
**Olfactory**	14.88 (n = 5)		

### The Modified Beer-lambert Law and the Effective Attenuation Coefficients for Highly Scattering Neural Targets

The full mathematical treatment of light travelling in biological tissue that absorbs and scatters light-waves (or optical photons) is described by the Radiative Transport Equation (RTE) [11], [12].
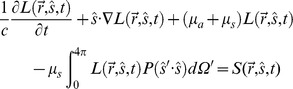
where 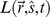 is the radiance *(W m^−2^ sr^−1^)* of the propagating light-wave; *µ_a_* and *µ_s_* are the absorption and scattering coefficients (*m^−1^*) of the biological tissue; 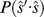 is the phase function describing the probability of a photon scattered to the radiation direction 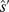 from its original radiation direction 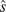;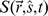 is the optical energy density *(Wm^−3^ sr^−1^)* generated in the biological tissue; *c* is the speed of light in vacuum and Ω is the solid angle.

The RTE is a complex equation, which has no analytical solution, since 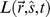 depends on both the spatial coordinate (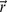), the radiation direction (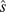), and time (*t*), resulting in a function with seven independent variables. 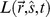
can be evaluated computationally with the RTE but requires an involved computational algorithm such as a Monte-Carlo stochastic simulation [13], [14]. Therefore, to extract quantitative parameters from our empirical measurements, a simplification of the RTE is needed. For most biological samples, including the brain, the scattering coefficient at the wavelengths tested here is typically one to two orders of magnitude higher than the absorption coefficient (*µ_s_>>µ_a_*). In addition, the phase function 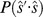
can be approximated by the Heyney-Greenstein function [15]:
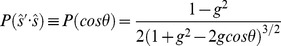
where *g* is the anisotropy factor and is generally assumed to be larger than ∼0.9 (0.9≤ *g* ≤1) in most biological tissues, indicating that the scattering light is predominantly forward-scattered. Under these conditions, the RTE can be approximated by the diffusion equation (the details of the simplification can be found in [11]):



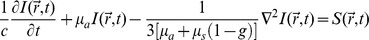
where 
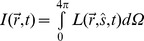
 is the irradiance (*Wm^−2^*), or in the laboratory commonly (but erroneously) called intensity of the light wave, and 
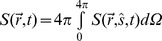
. To further simply the diffusion equation, we further assume that the optical propagation is in a steady-state condition (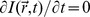) and there is no light being generated in the biological tissue (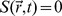). Therefore, the 1D diffusion equation can simply be written as a 1D second-order differential equation [12]:



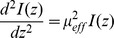
where 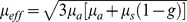 is the effective attenuation coefficient. Hence, the solution of the 1D diffusion equation is the modified Beer-Lambert Law [12]:



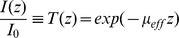
with *I_0_* being the irradiance measured at the fiber output of the optical fiber, and *z* being the longitudinal distance from the fiber output. The ratio of *I(z)* against *I_0_* is the optical transmittance *T(z)* which follows an exponential decay against the longitudinal distance *z*. In this study, we attempt to estimate the optical penetration depth within a brain nucleus to determine optical fiber source excitation efficacy of the opsin expressed within this neural target. It is clear from the above equation that it is not necessary –at the three wavelengths tested here- to individually measure *µ_a_, µ_s_* and *g*. Rather, simply measuring the effective attenuation coefficient *µ_eff_* is sufficient for this estimation.

### The Brain Atlas, and a Technique of Mapping the Effective Attenuation Coefficients across the Entire Brain

The technique of using an optical fiber to punch through a brain slice allows to collect data from well identified brain areas at very precise depths. However, it would be impractical to use this technique to map the effective attenuation coefficients 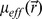 of many (i.e. hundreds) of brain areas, which would be required to obtain a quantitative picture across the entire brain. However, imaging brain slices using bright-field light transmission microscopy with monochromatic light and combining these images with the measured effective attenuation coefficients obtained from the punch-through method on selected neural targets allowed us to calculate and map out the effective attenuation coefficients across the entire brain. Whole brain slice imaging was performed on an Olympus VS 120 microscope, using transmitted light filtered via 546/20 nm band-pass filter and a 10x (N.A. 0.40) objective. To allow seamless, quantitatively correct tiling of multiple images of a single brain section, the manufacturer calibrated the microscope to normalize for uniform illumination and data acquisition across the entire imaging area. With this normalization, the illumination irradiance *I_0_* can be assumed to be a constant across the whole brain slice scan.

The illumination irradiance *I_0_* of the microscope is difficult to measure directly, instead brain slices containing the brain areas measured previously with the punch-through method were used to quantify and normalize *I_0_*_._ For a previously measured brain area, using the modified Beer-Lambert law, the illumination irradiance *I_0_* can be estimated by
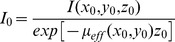



Effective attenuation coefficients *µ_eff_ (x, y)* of other brain areas not measured with the punch-through method can subsequently be calculated using
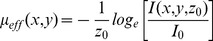



### The Effective Excitation Distance for Optogenetic Proteins

In optogenetic experiments, it is important to estimate the minimum optical irradiance required to effectively excite the desired neural area longitudinally to maximize excitation of the optogenetic proteins. Assuming the minimum excitation irradiance threshold for an optogenetic protein is *I_min_* and the irradiance at the fiber output is *I_fiber_*, the effective excitation distance d in the longitudinal direction of a neural target, which has an effective attenuation coefficient of *µ_eff_* can be calculated by the following equation
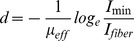



## Results

The most common approach to bring light into deep brain areas *in-vivo* is via optical fibers that are stereotactically placed above the brain area of interest. Our experimental approach of advancing a light emitting optical fiber through brain tissue modeled such a situation well, and enabled us to precisely determine light intensity at any depth along the longitudinal axis with respect to the fiber tip.

### Light Intensity Decreases Exponentially in Brain Tissue

An acute brain slice was placed into a perfusion chamber under an inverted microscope, and an optical fiber attached to a LED was placed directly on the tissue surface. Light emitted from this fiber propagated through the slice and was then collected by the objective and the chip of the attached monochromatic camera. A sketch of this configuration is shown in fig. 1A, and an example of an original image acquired with this setup is shown in fig. 1C. This configuration of imaging the light emitted from an optical fiber tip after it passed through a piece of brain tissue of known origin and of a known thickness *d* allowed effective measurement of the remaining light intensity at tissue depth *d*. From this value we could then calculate the ratio of the intensity of remaining light at depth *d* over the original light intensity at the optical fiber tip.

Subsequently, the optical fiber was lowered into the section in 5 µm steps, and similar images were acquired with each step. Advancing the fiber into the tissue (referred to as the “fiber punch-through method”), and taking repeated images at various depths effectively created a dataset of light intensity measurements in brain tissue at points progressively closer to the fiber tip. An alternative approach would have been to cut brain slices of different thicknesses and measuring light transmitted through each one of these slices. However, the fiber punch-through method allowed us to control for tissue depth (thickness) much more precisely than cutting sections of various thicknesses would have allowed us to do, and furthermore allowed us to measure the *exact* same piece of tissue at different depths.

From the measurements obtained at various tissue depths, light intensity ratios were calculated and plotted. Curve fitting indicated that the data points were best described by a single exponential function. An example of such a fit is shown in Fig.1B, representing a set of measurements with blue light (453 nm) recorded from a section of PPT. The ‘+’ symbols represent the measured luminance at each tissue thickness, and the superimposed line represents the exponential fit.

### Light Scattering Properties Vary across Different Brain Regions

**Figure 2:**
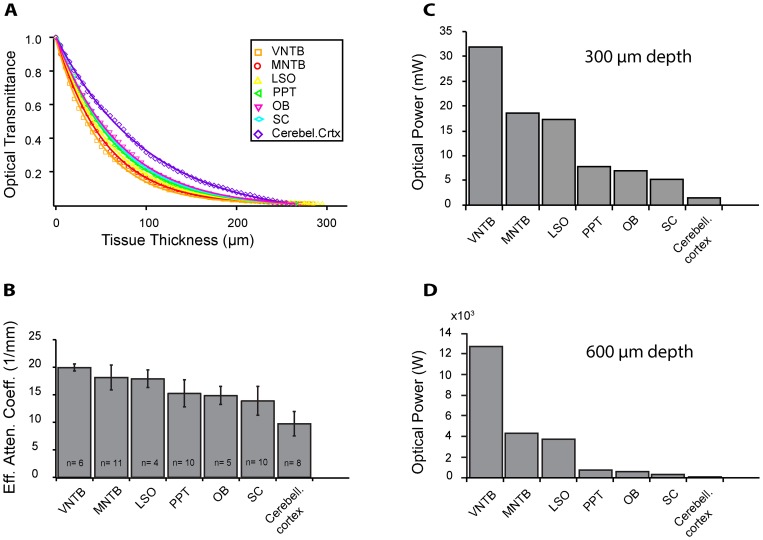
Optical transmittance through different types of brain tissue. 2A: Measurements using the fiber punch-through technique were taken in seven different brain areas with blue (453 nm) light. In each case, optical transmittance decreased exponentially with tissue thickness; however, the exponential decreases observed varied greatly with the type of tissue. Single measurements are represented by the respective symbols while the solid lines represent exponential fits of the data. 2B: Effective attenuation coefficients with SEMs for the seven brain areas: VNTB 19.96+/−0.26; MNTB 18.16+/−0.69; LSO 17.92+/−0.80; PPT 15.26+/−0.78; OB 14.88+/−0.74; SC 13.91+/−0.83; Cerebellum 9.76+/−0.78; all units are 1/mm. 2C: Optical power values that would need to be fed into a 100 µm diameter optical fiber when 300 µm of tissue needs to be illuminated at intensities typically used for Channelrhodopsin activation. 2D: Same as figure C except that in this example the illumination was calculated to hypothetically activate Channelrhodopsin over a distance of 600 µm from the fiber tip.

Seven different brain regions were measured with 453 nm light in the same way as the PPT shown above (Fig. 2A). The data points (colored symbols) were plotted against the distance from the fiber tip, and the set of measurements from each brain area were fitted with a single exponential function (colored lines). The results indicate that light intensity dropped at least 10-fold within a 200 µm distance from the tip of the optical fiber in each brain region tested. Importantly, the data suggest that this drop differs substantially among the brain regions tested. To systematically examine these differences, we calculated effective attenuation coefficients from the data (Fig. 2B). Average coefficients ranged from 19.96+/−0.26 for VNTB tissue, representing the lowest light transmittance of any region tested, to 9.76+/−0.78 for cerebellum, representing the highest transparency of all brain regions tested. (for all values and SEMs, see figure 2B and corresponding figure caption). Figures 2C & D shows the practical consequences of these differential coefficients on light penetration through the different types of tissue. Figure 2C plots the optical power required to illuminate neurons up to a tissue depth of 300 µm below the optical fiber tip with a light intensity of at least 10 mW/mm^2^ (the light power required for ChR2 activation 8–12 mW/mm^2^ [1]). To achieve this goal in cerebellar cortex, about 1.5 mW need to be emitted from the tip of the optical fiber, while in the case of VNTB, about 20 times as much optical power is required to achieve the same goal. Due to the nonlinear nature of light distribution in tissue, these differences become more dramatic for deeper penetration. For example, doubling the illumination depth from 300 µm to 600 µm would require about 20x the light intensity in the case of cerebellar cortex tissue (28 mW). By contrast, illuminating 600 µm of VNTB tissue to the same degree would require 12 W of light intensity, or 400 times the intensity required to illuminate 300 µm. These calculations suggest substantial differences in light scattering among different brain areas, and make the point that certain manipulations are possible in some brain areas but challenging in others.

### Light Scattering Varies with Wavelength

**Figure 3:**
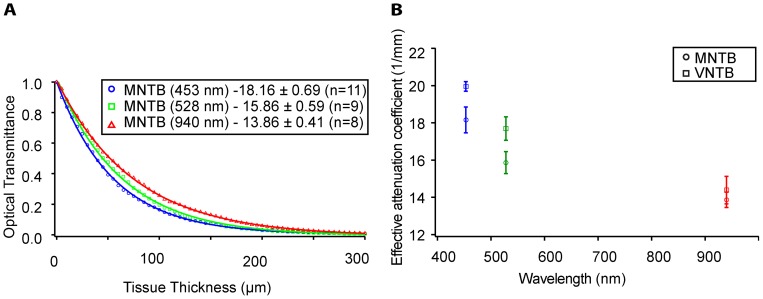
Effects of wavelength on optical transmittance. 3A: Optical transmittance in the MNTB as a function of tissue thickness and optical wavelength. The three color-coded data sets represent corresponding measurements with light of three different optical wavelengths (blue (453 nm), green (528 nm), and red (940 nm)). Longer-wavelength light penetrates tissue deeper, resulting in a higher transmittance at any given tissue thickness. 3B: Effects of light wavelength on transmittance in two brain areas (MNTB and VNTB). The effective attenuation coefficient decreases with wavelength for the three wavelengths tested. MNTB measurements are represented by round symbols while VNTB measurements are represented by square symbols. Measurements in the three different colors are indicated by the color-code of the symbols.

A traveling wave interferes with objects that are larger than its wavelength, but tends to bend around objects smaller than its wavelength. Thus, long wavelength light penetrates tissue deeper than short wavelength light. Since different light-sensitive molecules are optimally excited at a variety of wavelengths, we tested the influence of light wavelength on the penetration depth of light in brain tissue. Fig. 3A represents experiments in which MNTB was tested with three wavelengths: 453 nm, 528 nm, and 940 nm. As expected, the longest wavelength (940 nm) showed the most effective penetration, i.e. the smallest attenuation of light intensity with increasing distance from the fiber tip (red line), while the blue light (453 nm) attenuated within the shortest distance from the fiber tip (blue line). Similar observations were made for a second brain area (VNTB) that was tested in the same way (fig. 3B). Note that optical absorption cannot be neglected at all light frequencies (an assumption made for the three single light frequencies tested in this study), and thus there is no simple linear extrapolation between the points shown in figure 3B [16].

### Light Scattering Brain Atlas

**Figure 4:**
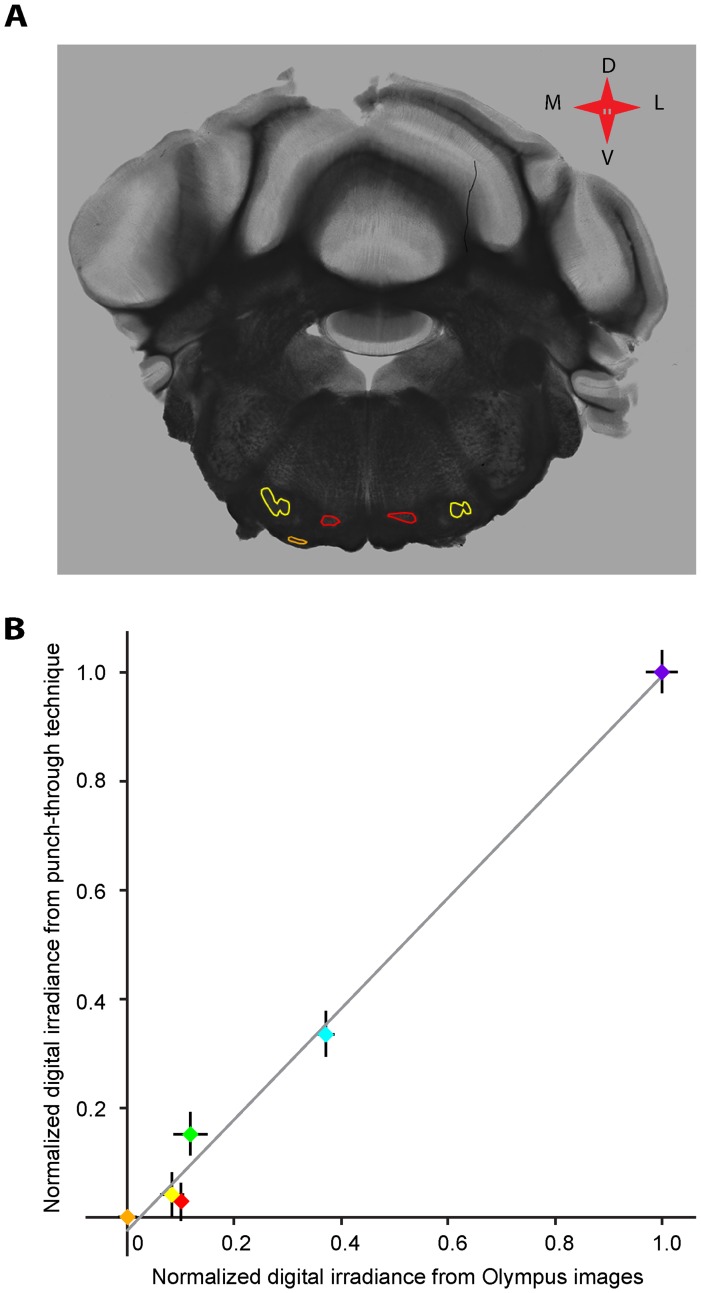
Relating fiber punch-though measurements to brain atlas measurements. 4A: Image of a 300 µm coronal section of mouse brain stem, taken on a calibrated virtual microscopy system with monochromatic light. Areas with higher optical transmittance appear brighter on the image, while areas with lower transmittance appear darker. MNTB, VNTB, and LSO are outlined in red, orange, and yellow, respectively. 4B: Correlation in digital irradiance for brain areas tested with both the fiber punch-through and the virtual microscopy method. Digital irradiance was measured in six brain areas (MNTB (red), VNTB (orange), LSO (yellow), PPT (green), SC (light blue), and cerebellum (dark blue) with both the fiber punch through and the virtual microscopy technique. Results were normalized and plotted against each other. Each colored symbols represents the measurements from one brain area with two methods, the solid line indicates complete overlap between the measurements. The bars attached to each data point represent the standard error.

While the fiber punch-through method allowed for measurements of light scattering properties in anatomically defined brain areas, it is a relatively slow method. Measuring many different brain areas with this technique would not be feasible. However, to extend usage of our data to other areas of the brain without the need for additional punch-through measurements, we prepared a brain atlas containing light scattering values from the entire mouse brain. For this atlas, sections of 300 µm thickness were prepared from mouse brains, and imaged with an Olympus virtual microscopy system (Olympus VS 120) using monochromatic transmitted light (546 nm band pass filtered with a 20 nm band-pass width). The resulting images consist of relative differences in tissue translucency in grey scale between different brain areas in the section. Fig. 4A shows an example of such an image, with several nuclei marked with colored lines on the section. For brain areas that were also measured with the punch-though method, the relative grey values of the images correlated very well with the effective attenuation coefficients measured with the punch-through method (Fig. 4B), suggesting that the grey values of the images can be used as a basis to calculate the effective attenuation coefficients for brain areas that have not been tested with the punch-through method. Importantly, the grey value images in combination with the effective attenuation coefficients measured with the punch-through method allowed us to establish an atlas of brain translucency that can be used to calculate the light scattering properties of any brain area in the adult mouse brain.

### Applying the Data to Experimental Design

An investigator planning an experiment involving light activation of a given protein *in-vivo* is typically interested in the amount of light required to activate the protein at a distance *d* from the fiber tip. In order to correctly determine the required amount of light, the following parameters must be considered: 1) The wavelength of the light, 2) the largest distance from the fiber tip at which proteins are to be activated, 3) the specific light scattering properties of the brain area involved, and 4) the diameter of the fiber tip. We produced a computer program that calculates the required amount of light for a given experiment based on user input of these parameters. The program also incorporates the brain atlas described above. For further information, see www.optogeneticsapp.com.

## Discussion

### Main Findings

There are three main findings of this study: 1) Light emitted into brain tissue from a point source such as an optical fiber declines exponentially in intensity with increasing distance from the fiber tip 2) There are substantial differences in light scattering properties among different brain areas, resulting in a need for specific knowledge about any given brain area to be illuminated 3) The light wavelength used in a particular experiment additionally influences the scattering properties, with longer wavelength light penetrating deeper into the tissue. The results obtained in this study could be integrated into a brain atlas of light scattering in the mouse brain, as well as a computer program that allows a user to easily determine the light requirements for any given experimental situation.

The most important finding is the observation that there are substantial differences in the optical properties across different brain areas. For simplicity, previous studies have assumed that light propagation through brain tissue is similar throughout the brain, and have calculated the light requirements for optogenetic experiments with a single effective attenuation coefficient [8]–[10]. Our results show that a differential approach is needed, because the observed differences in effective attenuation coefficients can have substantial consequences on experimental design. Figure 2C & D illustrates this point and suggest that certain manipulations are possible in some brain areas but not others.

In some experiments, one might want to restrict the volume of illumination, e.g. if an opsin is widely expressed in the brain [17] but only a certain region is to be manipulated with light. Thus, specific knowledge of the light requirements for a given experimental situation can inform an optimal experimental design, and this includes knowledge about the specific light scattering properties of the brain area to be manipulated, as well as knowledge about how different light wavelengths will affect the illumination. The data presented here aid with the experimental design of light delivery to deep brain areas, i.e. help an investigator estimate the correct amount of light required to obtain the desired illumination levels of deep brain areas. However, successful optogenetic manipulation also depends on parameters other than light delivery. For example, the expression levels of optogenetic protein needs to be sufficient to activate/suppress the specific neurons to be manipulated, and the specific membrane properties of the neurons to be manipulated will affect successful optogenetic activation/suppression of the neurons. Thus, successful light delivery to the brain areas to be manipulated is only one component of successful optogenetic manipulation.

### Comparison with Previous Studies

Aravanis and colleagues [8] first characterized the optical scattering effect in mouse brain cortical tissue by measuring the optical attenuation at different slice thicknesses. In their work, the Kubelka-Munk model (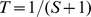
where *S* is the scattering coefficient) was used in their data fitting. However, our measurements were best fitted with an exponential function and the data cannot be satisfactorily fitted with the Kubelka-Munk equation. The discrepancy mainly occurs at larger distances (*z* >200 µm), and our data show that light attenuates much faster than predicted by the Kubelka-Munk model, resulting in a much reduced excitation distance of neural targets in our results. More recently, Stark et al. [18] report that their measurements of optical attenuation at larger distances from the fiber tip cannot be well fitted with the Kubelka-Munk equation, although at shorter distances the data fit with the equation is good. The differences are likely due to the different optical detectors being used in these experiments. In our measurements, a single pixel of the CCD camera along the center of the propagation axis of the optical fiber was used to construct the optical transmittance curve. By contrast, the previous studies used a large area photodetector to measure the optical attenuation, which also collects light not strictly propagating along the optical axis. This difference could potentially result in differences in the data.

When light comes out of an optical fiber tip, light spread as well as the radius of light increases as light propagates further away from the fiber output. This cone shape of light propagation increases the beam area (*A*), thus reducing the optical irradiance of the light beam (*I = P/A*). However, this reduction of optical irradiance due to beam spreading from the optical fiber is much more gradual than the optical scattering in the brain tissue, so this beam spreading effect can be neglected or considered to be absorbed in the effective attenuation coefficient *µ_eff_*. For example, the numerical aperture (NA) of the 100 µm core (*r* = 50 µm) diameter optical fiber that was used in our measurement is 0.22, such that the acceptance angle of the light cone is 9.5 degrees (*NA = n sin^−1
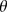
^*) where n≈1.33 in water). The radius of the beam increases over a propagation distance of *d* = 500 µm by 83 µm *(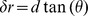
*); thus the beam area increases by a factor of 7, resulting in an optical irradiance reduction to 14% of its original output. At the same time, according to our measurements, the optical transmittance of the MNTB due to optical scattering after 500 µm of propagation is 0.01% at 453 nm wavelength. Therefore, we conclude that the optical fiber beam spreading is not a significant effect in estimating the optical irradiance in brain tissues.

### Application of the Findings to Future Experiments

One goal of this study was to provide a body of knowledge on light scattering properties of the mouse brain that could be used by investigators as a tool to optimize the light stimulation for a specific experimental situation. To this end, data from several brain areas were collected but the fiber punch-through method, while allowing us to obtain data in great detail, was not efficient enough to use for a multitude of brain areas. Therefore we resorted to virtual microscopy to image the entire mouse brain with monochromatic transmitted light. The resulting images consisted of gray-value pixels, which represented the differences in optical properties between these different brain areas. The differences in grey values between different brain areas obtained with virtual microscopy corresponded well with the differences observed in the fiber punch-through method, allowing us to calibrate the results from the two approaches to each other. Thus, we obtained data on the light scattering properties of the entire mouse brain, allowing an investigator look up the brain area of choice in the light scattering atlas, and determining the associated effective attenuation coefficient for that area. This coefficient can be entered into a computer program, together with information on the desired stimulation wavelength and volume of brain tissue to be illuminated super-threshold. The computer program then estimates the required light intensity at the optical fiber tip to meet the desired criteria. The use of these tools should allow an experimenter to design optogenetic manipulation *in-vivo* with better precision and more confidence that the brain area to be activated by light will actually be illuminated at a super-threshold intensity. Moreover, the delivered light can be adjusted to be super-threshold for opsin activation in the desired brain area, and fall to sub-threshold values at the borders of the brain area of interest, reducing unspecific activation of adjacent neuronal areas. For further information, see www.optogeneticsapp.com.
